# Potential of mesenchymal stem cells alone, or in combination, to treat traumatic brain injury

**DOI:** 10.1111/cns.13300

**Published:** 2020-03-10

**Authors:** Alison E. Willing, Mahasweta Das, Mark Howell, Shyam S. Mohapatra, Subhra Mohapatra

**Affiliations:** ^1^ Department of Neurosurgery and Brain Repair Center of Excellence for Aging and Brain Repair University of South Florida Morsani College of Medicine Tampa FL USA; ^2^ Department of Molecular Medicine University of South Florida Morsani College of Medicine Tampa FL USA; ^3^ James A. Haley Veterans Hospital Tampa FL USA; ^4^ Department of Internal Medicine University of South Florida Morsani College of Medicine Tampa FL USA

**Keywords:** clinical trials, combination treatment, stem cells, traumatic brain injury

## Abstract

Traumatic brain injury (TBI) causes death and disability in the United States and around the world. The traumatic insult causes the mechanical injury of the brain and primary cellular death. While a comprehensive pathological mechanism of TBI is still lacking, the focus of the TBI research is concentrated on understanding the pathophysiology and developing suitable therapeutic approaches. Given the complexities in pathophysiology involving interconnected immunologic, inflammatory, and neurological cascades occurring after TBI, the therapies directed to a single mechanism fail in the clinical trials. This has led to the development of the paradigm of a combination therapeutic approach against TBI. While there are no drugs available for the treatment of TBI, stem cell therapy has shown promising results in preclinical studies. But, the success of the therapy depends on the survival of the stem cells, which are limited by several factors including route of administration, health of the administered cells, and inflammatory microenvironment of the injured brain. Reducing the inflammation prior to cell administration may provide a better outcome of cell therapy following TBI. This review is focused on different therapeutic approaches of TBI and the present status of the clinical trials.

## 1 INTRODUCTION

The Centers for Disease Control and Prevention defines a traumatic brain injury (TBI) as a disruption in normal brain function as a result of any blow to the head.^1^ It is a major health concern in the United States and around the world. According to the Health United States Report 2016, 2.8 million people in this country sustain this injury annually, and it is estimated that of these, ~50 000 die, ~282 000 are hospitalized, and the remaining 2.5 million (or 89%) are treated and released from the emergency department.^2^ Long‐term disability depends on the severity of the TBI,^3^ the presence of diffuse axonal injury on imaging,^4^ and the intensity of neurorehabilitation.^5^ Further, recovery may take an extended period of time^6^ and the patient may be left with neurobehavioral deficits including mental health disorders such as depression, anxiety or psychotic disorders, cognitive disorders related to executive functioning, and aggression.^7^ In a prospective study that followed TBI patients for up to 1 year, the distribution of mild, moderate, and severe TBI was comparable to what is observed in the real‐world population with 49% having mild TBI, 34% having moderate TBI, and 17% having a severe injury. About half of the study population did not return to their previous work after 1 year, and ~28% never returned to work of any kind.^8^ Also, long‐term disability is seen occasionally even in those with mild TBI.^9^ Thorough reviews of TBI epidemiology have recently been published.^10^, ^11^

Thus, while TBI is a significant public health problem, unfortunately there is no single therapy that has proved efficacious in its treatment. Similar to the situation with other brain injuries (such as the failure of neuroprotective glutamate receptor antagonists and antioxidant treatments in clinical trials for stroke^12^, ^13^) and neurodegenerative diseases, there have been myriad‐positive preclinical studies in TBI models and all of these promising therapies have failed in clinical trials. Various reasons have been advanced for these failures, including, but not limited to, differences in brain anatomy and physiology between rodents and humans, inadequate animal models, failure to test the treatment in a clinically relevant way coupled with failure to remain faithful to the preclinical testing parameters in the clinical trials, underpowered studies, heterogeneity of TBI injury, and insensitive outcome measures in both preclinical and clinical studies. There is no dearth of discussions in the literature identifying these shortcomings in the therapeutic development and testing of potential new treatments for TBI.^14^, ^15^, ^16^, ^17^, ^18^

What we are left with for treatments is a general approach that is akin to crisis management. According to the current Brain Trauma Foundation Guidelines, based on the best available medical evidence for the management of severe TBI, it is imperative to provide adequate nutrition, support breathing by tracheostomy, and perform a large decompressive craniectomy.^19^ The underlying problems for developing an effective treatment for TBI are 2‐fold. First, the injury can be unique to the patient, depending on the type of TBI and the region of the brain affected. Second, once that injury occurs, a complicated neurodegenerative cascade is triggered; resolving any one of these pathological processes is not enough to prevent or terminate the others. In this review, we will discuss the pathophysiology of TBI with emphasis on immune and inflammatory function. We will also discuss the evidence for the development of a mesenchymal stem cell (MSC)‐based treatment that can suppress immune and inflammatory/degenerative cascades and provide neuroprotection.

## 2 PATHOPHYSIOLOGY OF TBI

The pathophysiologic mechanisms of TBI are poorly understood as the anatomy of the brain is uniquely complex with multiple cell types (neurons, astrocytes, oligodendrocytes, and microglia) and multiple subtypes of these cells. While we now know that neural stem cells (NSC) exist within the adult brain and some degree of axonal, dendritic, and synaptic plasticity occurs, we have yet to fully exploit the brain's regenerative capacity to repair an injury. Add to this the complicated neuronal networks throughout the brain and neural repair is a daunting task. Ramon Y Cajal, widely regarded as one of the fathers of modern neuroscience, stated in his treatise on neural development and regeneration “…once development was ended, the founts of growth and regeneration of the axons and dendrites dried up irrevocably. In the adult centers the nerve paths are something fixed, ended and immutable. Everything must die, nothing may be regenerated. It is for the science of the future to change, if possible, this harsh decree*.*”^20^ When we consider additional factors such as the confined space within the skull that contributes to increased intracranial pressure as edema develops and the blood‐brain barrier (BBB), which can make therapeutic access to the brain difficult, the task of repairing the brain after TBI may seem insurmountable.

The pathophysiology of TBI occurs in two main phases—the primary insult and the secondary sequelae. The primary insult is the initial blow to the head. It can be a penetrating wound or a closed‐head injury. The nature of the injury can be focal, involving a very circumscribed area of the brain, or it can be more diffuse causing widespread axonal injury. Then, there is the special case of the coup‐countercoup injury as the brain impacts the skull in two sites on opposite sides of the brain; this is observed with trauma that involves deceleration of the head. Depending on the nature of the trauma, neurons, astrocytes, and oligodendrocytes may be destroyed, bleeding may occur, axons may be severed, and a contusion may form.^21^ The immediate pathological consequences of these injuries are similar to those observed with cerebral ischemia—excitotoxicity, changes in ion flux (Ca^++^, Na^+^, and K^+^) across the cell membrane, loss of ATP, lactate production, induction of cortical spreading depression, cytokine production, and loss of barrier function at the BBB.^22^, ^23^, ^24^

The severity of the initial blow and the immediate pathophysiologic changes that occur determines the severity of any subsequent secondary degenerative processes. In the acute period, neurons and axons continue to die, and there is damage to the vascular endothelium; this, in turn, allows blood components to leak into the brain parenchyma, including peripheral immune cells, which then contribute to the pro‐inflammatory environment. Astrocytes swell and tissue edema occurs. If edema is not controlled, intracranial pressure increases, which can lead to compression of arteries and decreased cerebral blood flow; cerebral ischemia commonly occurs under these conditions leading to a vicious cycle of increasing edema, increasing intracranial pressure, and increased ischemia that can lead to death.

## 3 INFLAMMATION IN TBI

The main architects of the local inflammation at the site of injury are the microglia. Once a TBI has occurred, it is the microglia that proliferate and migrate to the site of the damage. As these microglia work to remove the cellular debris at the lesion site, they produce cytokines and chemokines that activate pattern recognition receptors to bind damage‐associated molecular patterns, and attract and polarize peripheral immune cells. The first peripheral immune cells into the damaged tissue are neutrophils^25^ followed 24‐48 hours later by monocytes or macrophage, and T cells all of which are releasing cytokines and chemokines. Once the peripheral immune cells have established a strong pro‐inflammatory response in the brain in the acute to subacute stage of TBI, then tissue damage is likely to be exacerbated. Further, the T cells may become activated through antigen presentation by microglia and macrophage, mobilizing the adaptive immune system^26^ and potentially leading to autoimmunity.^26^, ^27^

In addition to local neuroinflammatory processes within the brain after TBI, systemic immune and inflammatory processes are also impacted and have recently been reviewed.^28^, ^29^ Peripheral immune cells are mobilized from the bone marrow,^30^ thymus,^31^ and spleen^31^ early after the TBI. While there is a brief recovery in thymocytes and classical monocytes in thymus and spleen within the first 2 weeks postinjury, over time these cell populations decline again and may be responsible for the post‐TBI immune suppression that is observed in patients.^29^, ^31^ Also implicated in the peripheral immune response and long‐term immune suppression is both the activation of the hypothalamus‐pituitary‐adrenal axis, through the release of glucocorticoids and the sympathetic nervous system, through release of catecholamines.^29^, ^32^

Ideally, inflammation will be a self‐limiting process. There are endogenously formed products of arachidonic acid metabolism that actively inhibit pro‐inflammatory responses.^33^ These include the lipoxins, resolvins, protectins, and maresins, which decrease pro‐inflammatory cytokine secretion,^34^ alter migratory signals for peripheral immune cells,^35^ and stimulate neuroprotective and tissue regeneration processes.^36^ There has been very little research on these resolving mediators in TBI, but administering the lipoxin, LXA4, into the lateral ventricles 10 minutes after a TBI induced by weight drop, reduced pro‐inflammation, BBB disruption, and lesion size.^34^ Further, its receptor, ALX/FPR2, is upregulated in astrocytes.^37^

Unfortunately, in many instances inflammation may not resolve and becomes chronic.^38^ In the aged brain, there is increased recruitment of peripheral macrophage into the TBI brain.^39^ In addition to cellular infiltration of pro‐inflammatory immune cells after TBI, sustained complement C3 activation leads to chronic inflammation by activating microglia and astrocytes in the region around the initial lesion and contributes to further neuronal loss 30 days post‐TBI.^40^ Using a combination of magnetic resonance imaging, magnetic resonance spectroscopy, and positron emission tomography in a rat lateral fluid percussion model, inflammation was shown to be present still 6 months post‐TBI.^41^ Even 12 months after a controlled cortical impact in mouse, there is increased immunolabeling for IBA1 (microglia) and glial fibrillary acidic protein (GFAP).^42^ What's more, this chronic inflammation is associated with continued behavioral deficits.

## 4 EXPERIMENTAL PHARMACEUTIC TREATMENTS FOR TBI

### 4.1 Pharmaceuticals

The focus of current pharmacological interventions after TBI is to manage level of consciousness, neuropsychiatric, neurocognitive, and neurobehavioral symptoms that may arise.^43^ With the occurrence of so many interconnected neuro‐immune‐inflammatory pathologic cascades engaged after TBI, it is not surprising that therapies targeting one specific degenerative pathway have failed to demonstrate efficacy in clinical trials. Drug interventions that have been studied can generally be categorized by their therapeutic target. One class of drugs are those that prevent calcium ion flux. An example of such an approach is the calcium channel blocker, Nimodipine, which showed promising effects in rodents but exhibited only a small effect on TBI patients.^44^, ^45^ Disruption of intracellular calcium signaling may also improve outcome after TBI. Cyclosporin is a T‐cell immunosuppressant that acts by binding to cyclophilin; the cyclosporine‐cyclophilin complex binds to calcineurin preventing dephosphorylation of NFAT, translocation to the nucleus, and increased transcription of interleukin (IL) 2.^46^ In the absence of cyclosporine, calcineurin is regulated by calcium and calmodulin. Cyclosporin prevents calcium ion transport into the mitochondria in animal models but not in TBI patients.^47^

There are also a number of studies that have targeted excitotoxicity, specifically glutamate release and overstimulation of the NMDA receptor. For example, Selfotel is a NMDA antagonist and the first glutamate antagonist to enter into Phase III clinical trial. This trial was discontinued because of high mortality and a failure to improve Glasgow Outcome Score.^48^ The results from clinical studies of other NMDA receptor antagonists also failed to demonstrate any efficacy of treatment.^49^ While not a true antagonist, magnesium blocks the NMDA receptor calcium channel. Increasing available magnesium also had no effect on TBI outcome.^50^

Another approach has been to target oxidative stress produced by oxygen radical formation and lipid peroxidation. The lipid peroxidation inhibitor, Tirilazad mesylate, which is an approved drug in Europe to treat aneurismal subarachnoid hemorrhage, showed promising neuro‐ and vaso‐protective responses in animal models of moderate‐to‐severe TBI but failed to show improvement over placebo control in Phase III clinical trials involving human TBI patients.^51^, ^52^ Pegylated superoxide dismutase, a free radical scavenger, was found to be effective in preventing secondary injury in preclinical and Phase I clinical studies but failed to show reduction in mortality or improve neurologic outcome in Phase III trials.^53^, ^54^ Another antioxidant that may be promising is *N*‐acetylcysteine. When administered to patients within 24 hours of mild TBI, symptoms were significantly better compared to a placebo‐controlled group.^55^

Another target that has been examined is the treatment of inflammation with corticosteroids, statins,^56^ cannabinoids, and bradykinin B2 receptor antagonists.^50^ In addition, the gonadal hormones, estrogen and progesterone, both showed promising results in preclinical studies but failed to show beneficial effects in clinical trials.^57^, ^58^, ^59^

The more recent approach has been to search for potential therapies that target more than one pathway. One such strategy is to use a pharmacologic that interacts with multiple receptor types, which, thereby, produces more than one effect. For example, sigma receptor agonists selective for either sigma 1 (σ1) or σ2 receptors (or both) have both neuroprotective and anti‐inflammatory effects in rodent models of stroke.^60^ More recently, σ1‐selective agonists have been shown to decrease neuroinflammation,^61^ while σ2‐selective agonists are neuroprotective after TBI.^62^

### 4.2 Combination drug treatment regimens for TBI

In an effort to increase treatment efficacy, multiple drug combinations have been administered together to target multiple neurodegenerative pathways. Based on reported success in the treatment of HIV/AIDS with HAART,^63^ recently a combination drug therapy was designed for the treatment of TBI^64^ combining vitamin D3, progesterone, omega 3 fatty acids, and glutamine administration for the first 72 hours for TBI patients with a poor prognosis; all patients improved beyond original expectations. However, these case studies included only three patients, there were no controls, and a larger study has not been performed to validate these observations. Another example of a combination of drug therapy is the progesterone and 1,25‐dihydroxyvitamin D3 combination, which was effective in reducing neuroinflammation as compared to treatment with the drugs separately.^65^, ^66^

## 5 CELL THERAPY IN TBI

A number of different cell types have been examined as potential therapeutics for TBI. The first studies in the field focused on replacing neurons in order to rebuild the neural circuitry. The earliest cells examined were already postmitotic neurons,^67^, ^68^, ^69^ and there was variable therapeutic success. Gradually, these studies were replaced by studies using stem cells. In a mouse model, NSC survived, differentiated, migrated to the lesion site, and improved motor and cognitive function after TBI.^70^ Also, embryonic stem cells were shown to improve functional outcome after TBI in rodents, but tumors also formed.^71^ Later studies involved predifferentiating stem cells into more lineage‐restricted precursors in order to reduce the likelihood of tumorigenesis.^72^, ^73^ In an effort to reduce reliance on embryonic or fetal tissue, there have been a number of studies more recently focused on induced pluripotent stem cells derived from adult somatic cells.^74^, ^75^, ^76^

In addition to these embryonic and NSC for which there are direct developmental pathways to produce neurons, astrocytes, and oligodendrocytes, another source of stem cells that has received a great deal of attention is MSCs. Originally, MSCs were isolated from bone marrow where they support hematopoiesis. However, it has become clear that MSCs reside in many tissues in the body,^77^ which may explain why they appear to be efficacious for treating so many different injuries and diseases. Because of their pleiotropic characteristics, these cells have significant therapeutic potential for various diseases including TBI. Following administration, MSCs have shown to penetrate the BBB, migrate to the site of injury, and secrete several growth factors including brain‐derived neurotrophic factor (BDNF), glial‐derived neurotrophic factor (GDNF), vascular endothelial growth factor (VEGF), nerve growth factor (NGF), and regenerate BBB and neuronal and glial tissues.^78^, ^79^, ^80^, ^81^ MSCs also modulated inflammation by inhibiting interleukin six (IL‐6) and IL1‐β and enhancing IL‐10.^82^ The anti‐inflammatory effect of MSC was reported in a study involving combined administration of MSC and NSC that led to increased recovery from stroke‐induced cerebral damage in rats as compared to MSC or NSC alone.^83^ Early studies demonstrated that intravenous,^84^ intraarterial,^85^ and intracerebral 86 administration of these cells improved motor and neurological outcome after TBI. Other methods of MSC delivery that have been used since include intranasal,^87^, ^88^, ^89^ intrathecal,^90^ and intracisternal.^91^ The postulated mechanism of repair in these early studies was transdifferentiation of the cells into neural cells. A number of studies were able to demonstrate that some of the transplanted MSCs expressed neuronal and astrocytic markers in vivo, but few cells survived.^84^, ^85^, ^92^, ^93^, ^94^ The results of in vitro studies were more positive^95^; however, the transformation from MSC to mature neuron occurred within hours,^96^ was reversible when the initiating stimuli were removed, and occurred in the presence of protein synthesis inhibition.^97^ It has been suggested that the “transdifferentiation” of MSCs into neurons requires MSCs to be in a toxic, stressful environment.^96^, ^97^ However, the ultimate proof that the cells transdifferentiate into neurons is still lacking; these “neurons” have never been shown to produce an action potential.^98^

In the ensuing years, several other putative mechanisms of repair for these cells have been studied. Thus, it has been suggested that MSCs may induce brain repair through the production of trophic factors or stimulating release of trophic factors from endogenous cells.^99^, ^100^, ^101^ These paracrine mechanisms of repair have recently been reviewed in some detail.^102^, ^103^, ^104^ More recently, MSC‐derived exosomes have been examined as the paracrine source of neuroprotection and anti‐inflammation. These exosomes improved cognitive function and reduced inflammation as determined by decreased IL‐1 and increased IL‐10 in the brain after TBI.^105^ Later studies are consistent with these results.^106^, ^107^ MSC‐derived exosomes have also been tested in porcine model of TBI coupled with hemorrhagic shock; consistent with the rodent data, the pigs had fewer cognitive deficits as determined with a neurological severity score and recovered faster than nontreated pigs.^108^ There is also evidence for MSC inducing immune suppression^109^, ^110^ and anti‐inflammation^106^, ^111^, ^112^, ^113^; stimulating neurogenesis^88^, ^114^, ^115^ and angiogenesis^107^, ^116^, ^117^; activating survival pathways^118^ and inhibiting apoptotic pathways^101^, ^119^; and enhancing neuroplasticity through neurite outgrowth^120^, ^121^ and synaptogenesis.^78^, ^107^

### 5.1 Clinical translation of cell therapy for TBI

As described in the previous section, many different approaches have shown promise for treating TBI‐induced pathologies and stimulating tissue regeneration in animal models. However, none of these have thus far translated into therapeutic benefit in human patients and the early clinical trials have not used pure MSC. Table 1 shows the current comprehensive status of clinical trials of stem cell therapy for TBI. The earliest clinical studies reported in the literature used bone marrow‐derived mononuclear cells (BMMNCs), which is a heterogeneous mixture of immune cells and stem cells including MSCs. The first major cell therapy trials for TBI using BMMNCs were conducted on children by Cox and colleagues in 2011.^122^ In the preclinical studies and Phase 1 clinical trial, treatment reduced BBB permeability and neuroinflammation after TBI. In a second pediatric study from the same research group, the TBI‐induced increase in intracranial pressure was reduced in the cell treatment group.^123^

**Table 1:** Current status of cell therapy clinical trials of traumatic brain injury (TBI)

NCT number	Title	Status	Interventions	Phases	Enrollment
NCT04063215	A clinical trial to determine the safety and efficacy of hope biosciences autologous mesenchymal stem cell therapy for the treatment of traumatic brain injury and hypoxic‐ischemic encephalopathy	Not yet recruiting	Drug: HB‐adMSC	Phase 1 Phase 2	24
NCT02525432	Autologous stem cell study for adult TBI (Phase 2b)	Enrolling by invitation	Biological: Placebo Infusion Biological: Autologous BMMNC Infusion	Phase 2	55
NCT01575470	Treatment of severe adult traumatic brain injury using bone marrow mononuclear cells	Completed	Biological: autologous bone marrow mononuclear cells	Phase 1|Phase 2	25
NCT02416492	A study of modified stem cells in TBI	Completed	Biological: SB623 cells Procedure: Sham Control	Phase 2	61
NCT01851083	pediatric autologous bone marrow mononuclear cells for severe traumatic brain injury	Active, not recruiting	Biological: autologous bone marrow mononuclear cells|Other: Placebo Infusion	Phase 1|Phase 2	47
NCT02959294	Use of adipose‐derived stem/stromal cells in concussion and traumatic brain injuries	Enrolling by invitation	Procedure: Microcannula Harvest Adipose|Device: Centricyte 1000|Procedure: Sterile Normal Saline IV deployment AD‐cSVF	Phase 1|Phase 2	200

There are also clinical trials of BMMNCs in the adults with severe TBI. In this population, treatment with BMMNCs resulted in structural preservation of the corpus callosum and corticospinal tract and these changes were correlated to neurocognitive outcomes; in addition, there was a reduction in the pro‐inflammatory cytokine response to injury (NCT01575470).^124^ A Phase 2 (NCT02525432) and Phase IIb (NCT02416492) follow‐up studies are currently underway.

Of the currently registered clinical trials specifically investigating MSCs, there are two. Hope Bioscience has a safety and efficacy clinical trial of its adipose‐derived MSCs. In another clinical trial (NCT02416492), the safety and efficacy of San Bio's proprietary adult bone marrow‐derived MSCs genetically modified to express the intracellular domain of human Notch‐1 to treat chronic TBI. Clinical trial of these cells in stroke patients demonstrated that the cells were safe and induced significant motor function improvement in adults according to European Stroke Scale, the NIH Stroke Scale, and the Fugl‐Meyer scale.^125^

### 5.2 Potential of MSC and anti‐inflammatory combination treatments for TBI

Because of inflammation and other ongoing neurodegenerative cascades, the brain environment post‐TBI is a hostile environment for transplanted cells. Without some adjunctive treatment, cell survival is limited. For NSC and neurons, where the cells are needed to rebuild neural circuitry, survival is critical. For MSCs, whether survival is necessary depends on where and how the cells are having their effects. The immune‐suppressive effects of the cells are systemic, so delivery to the brain is not critical.^113^, ^126^, ^127^ Their anti‐inflammatory effects are both systemic and local within the brain, so some cells need to enter the brain.^111^, ^112^ These functions have led to studies of these cells as cotransplants to enhance the survival of other cells; an example from the spinal cord injury literature demonstrated that MSCs cotransplanted with NSC in injured spinal cord resulted in increased survival of the NSC.^128^ While MSCs do have anti‐inflammatory properties, the noxious environment may decrease their survival as well,^129^ which may limit their neuroprotective effects. To deal with the problem of a toxic, degenerative environment in the brain post‐TBI, investigators have adopted multiple approaches to enhance cell survival.

One approach has been to delay transplantation so that the pathophysiology can stabilize; for example, in a study comparing MSCs with MSCs in a scaffold,^130^ MSCs were administered two months post‐TBI so that regenerative and repair physiologic processes would dominant in the parenchyma around the TBI lesion. Another approach has been to administer a drug treatment along with the cells or prior to cell transplantation. For example, Mahmood and associates administered MSCs intravenously 24 hours after TBI.^131^, ^132^ At the same time, statin treatment was started and continued for 14 days. Combination treatment with either atorvastatin or simvastatin improved recovery on the modified Neurological Severity Score (mNSS). In another study, investigators combined an early (1 hour post‐TBI) injection of the β adrenergic antagonist, propranolol, with an intravenous administration of MSCs at 72 hours post‐TBI.^133^ The underlying premise of the study was that propranolol decreases the TBI‐induced Sympathetic Nervous System hyperactivity; this decreased activity then helps to maintain cerebral perfusion, thereby decreasing post‐TBI ischemia and cell death. The MSCs were administered to manage the secondary inflammatory state. Unfortunately, the combined effects of propranolol and MSCs were not synergistic or additive; favorable outcomes (decreased serum norepinephrine, BBB permeability, microglial activation, cognitive function) could be achieved solely by the MSCs. In another study, investigators administered a calpain inhibitor 30 minutes after TBI and then transplanted the MSCs at 24 hours post‐TBI.^134^ There were significant decreases in pro‐inflammatory cytokines around the lesion, increased survival of the MSCs, and improvements on the mNSS.

We have taken a similar approach, but instead of modifying sympathetic activity in conjunction with targeting inflammation, we combined two inflammation‐modulating treatments. Based on earlier studies in which we identified chemokine (C‐C motif) ligand 20 (CCL20) as being significantly elevated after TBI,^135^, ^136^ we combined treatment with pioglitazone, a peroxisome proliferator‐activated receptor gamma agonist that inhibits CCL20, with treatment with MSCs.^88^ The pioglitazone was administered once a day for 5 days after TBI. On day 5, MSCs were administered intranasally. The combination of pioglitazone and MSCs was significantly better than either treatment alone on measures of anxiety, inflammation in the brain, and endogenous NSC proliferation. A similar approach was taken by Peruzzaro and associates when they engineered MSC's to overexpress IL‐10^137^ before transplantation in a TBI model.

Growth or trophic factor delivery in conjunction with cell administration is another approach that has been studied. These growth/trophic factors may favorably condition the environment in the TBI brain, or they may protect the transplanted cells from cell death. GDNF, epidermal growth factor, and VEGF have been shown to protect the brain from neuronal injury and increase regeneration of different cell types.^138^, ^139^, ^140^ However, growth factor delivery can be problematic. Growth factors have very short half‐lives, necessitating continual local delivery.^141^ Further, systemic delivery is often associated with side effects.^142^ As a result, it is imperative to deliver the growth factors into the brain near the injury. Liu et al^143^ showed that intracerebroventricular administration of fibroblast growth factor 2 (FGF2) for 7 days beginning at the time of MSC transplantation resulted in faster improvement in the forelimb placing and balance beam tests compared to the no treatment TBI group and the cell‐only group. Insulin‐like growth factor‐1 known to have a crucial role in MSC proliferation and putative differentiation to neuronal cells.^144^ This approach has shown to improve cell injury and motor activity of injured rats and improved metabolic and nutritional outcomes following TBI.^145^, ^146^ However, even growth factor delivery directly into the central nervous system (CNS) can lead to adverse effects. In a recent review of NGF trials for CNS diseases, the authors point out significant adverse effects such as pain and weight loss led to the discontinuation of the studies.^142^, ^147^

The most common procedure for combining a growth factor treatment with the delivery of MSCs has been to transfect the cells with specific growth factors. For example, overexpressing FGF21 in MSC resulted in improved performance on the Morris water maze, increased hippocampal neurogenesis and dendritic outgrowth.^148^ Other investigators have focused on the neurotrophins. Wu and associates overexpressed NT3 in MSCs and observed decreased glial activation, a smaller brain lesion, and decreased edema in the brain,^149^ while a number of investigators have focused on increasing BDNF expression in the cells, essentially improving functional outcome.^150^, ^151^

More recently, transplanting MSCs with a bioactive scaffold or biomaterial that secretes growth factors has been used to enhance survival, migration, and differentiation of transplanted cells.^152^ In one version of a scaffold, Bhang and associates suspended MSCs in a fibrin gel laced with FGF2.^153^ The MSCs in the fibrin glue with FGF2 decreased lesion size and apoptotic cell death more than MSCs in glue alone. When a functional peptide derivative of BDNF was incorporated in a self‐assembling hydrogel scaffold prior to seeding with MSCs and activated astrocytes, the resulting structure was able to reduce TBI lesion size.^154^

## 6 FUTURE PERSPECTIVES

Over the last two decades, there is a wealth of preclinical data, suggesting that MSCs may be an effective treatment, either alone or in combination, to improve outcome after TBI. Clinical trials are still in the early stages. Even with an abundance of data, there are still questions that should be addressed before these cells are routinely used to treat TBI or other CNS injuries or disease. Perhaps, most important is the issue of dosing. All studies in TBI to date have used a single dose of MSCs and doses used in the clinical studies have ranged from a total of 2.5 × 10^6^ cells up to 12 × 10^6^ cells per kilogram.^124^, ^125^ Not only do different research groups use different cell doses, in those studies using combination treatments, there is no indication whether dosing of the cell and/or the pharmacologic changes when the two are used in combination. To complicate issues further, patients may be taking a plethora of medications either prophylactically (such as baby aspirin or statins) or to treat common chronic diseases (beta blockers). For example, aspirin altered the immune and inflammatory profile of both endogenous monocytes harvested from stroke patients or healthy controls and MSCs in culture.^155^ When rats were treated with aspirin and cord blood‐derived MSCs singly or in combination, outcome tended to be somewhat worse with the combination treatment.^156^ These considerations are especially important as the population ages, since the elderly are at risk for TBI and polypharmacy is an issue in this population. In another example, when type I diabetic rats underwent a middle cerebral artery occlusion followed by MSC transplantation 24 hours later, the cells induced hemorrhagic transformation of the stroke and increased BBB leakage.^157^ The addition of niacin to the MSC therapy prevented these adverse effects.^158^ It is, therefore, essential that MSCs alone or in combination with other drugs must be tested for efficacy against a background of commonly prescribed drugs or medical conditions.

Another consideration with dosing is whether or not a single injection of cells is enough to maintain long‐term improvements in the functional outcome. As mentioned previously, TBI is accompanied by chronic inflammation.^38^, ^40^, ^41^, ^42^ As of yet, there are no studies in rodent models of TBI that have examined repeated administration of MSCs. There are indications from small clinical studies in other neurologic diseases or injuries that may hint at the potential efficacy of this dosing approach in TBI. For example, in patients with incomplete spinal cord injury, 30 × 10^6^ cells were administered into the subarachnoid space four times at three‐month intervals 159; while improvements in function were noted, the improvements were different for each individual patient. Patients with amyotrophic lateral sclerosis (ALS) were injected twice intrathecally with autologous MSCs (1 × 10^6^ cells/kg) 28 days apart and followed for 1 year.^160^ These patients had no severe adverse events and function as measured with the ALS Functional Rating Scale‐Revised stabilized.

Another issue that needs to be addressed in this field is the reliance on rodents in the early testing of potential new therapeutics. While using mice and rats is cost‐effective and allows manipulation of the genome, there are significant differences in the structure of the rodent brain and the human brain, not only in size, but also in, cerebrovascular volume, oxygen and glucose requirements, lissencephalic vs gyrencephalic architecture, and the amount of white matter present.^161^ It is imperative that larger animal models are used for the testing of new therapies including cell therapies so that we can distinguish good candidate therapies that are likely to succeed in clinical trials from those that will not. There has been some work in the development and characterization of porcine and ovine models of TBI, but there are currently no studies of MSC therapies and only a handful of MSC‐derive exosome studies^108^ in these larger animal models TBI models.^161^, ^162^

## 7 SUMMARY

The preclinical data on MSCs both alone and in conjunction with other treatment strategies are promising. These cells have entered at least Phase I (safety) clinical trials for multiple nervous system diseases and injuries, most notably cerebral ischemia,^163^, ^164^, ^165^, ^166^ multiple sclerosis,^167^, ^168^, ^169^, ^170^, ^171^, ^172^, ^173^, ^174^ Alzheimer's disease,^175^ and TBI.^133^, ^150^, ^176^ While we have a better understanding of the pathologic cascades triggered after TBI and the mechanisms by which MSCs repair the brain and improve functional outcomes, we are still years away from realizing an effective regenerative medicine therapy for TBI that is widely available to patients.

## CONFLICT OF INTEREST

The authors declare no conflict of interest.
